# Unilateral Condylar Hyperplasia: A 3-Dimensional Quantification of Asymmetry

**DOI:** 10.1371/journal.pone.0059391

**Published:** 2013-03-27

**Authors:** Tim J. Verhoeven, Jitske W. Nolte, Thomas J. J. Maal, Stefaan J. Bergé, Alfred G. Becking

**Affiliations:** 1 Department of Oral and Maxillofacial Surgery, Radboud University Medical Centre, Nijmegen, The Netherlands; 2 Department of Oral and Maxillofacial Surgery/Oral Pathology, VU University Medical Centre and Academic Centre of Dentistry, Amsterdam, The Netherlands; 3 Department of Oral and Maxillofacial Surgery, Academic Medical Centre/Emma Children Hospital Amsterdam, Amsterdam, The Netherlands; Glasgow University, United Kingdom

## Abstract

**Purpose:**

Objective quantifications of facial asymmetry in patients with Unilateral Condylar Hyperplasia (UCH) have not yet been described in literature. The aim of this study was to objectively quantify soft-tissue asymmetry in patients with UCH and to compare the findings with a control group using a new method.

**Material and Methods:**

Thirty 3D photographs of patients diagnosed with UCH were compared with 30 3D photographs of healthy controls. As UCH presents particularly in the mandible, a new method was used to isolate the lower part of the face to evaluate asymmetry of this part separately. The new method was validated by two observers using 3D photographs of five patients and five controls.

**Results:**

A significant difference (0.79 mm) between patients and controls whole face asymmetry was found. Intra- and inter-observer differences of 0.011 mm (−0.034–0.011) and 0.017 mm (−0.007–0.042) respectively were found. These differences are irrelevant in clinical practice.

**Conclusion:**

After objective quantification, a significant difference was identified in soft-tissue asymmetry between patients with UCH and controls. The method used to isolate mandibular asymmetry was found to be valid and a suitable tool to evaluate facial asymmetry.

## Introduction

Unilateral Condylar Hyperplasia (UCH) is a rare disorder that has been researched and discussed in numerous publications. Uncertainty exists about the aetiology. The condition is characterized by an asymmetry in the lower part of the face, due to persistent or renewed activity resembling growth in one of the mandibular condyles [1]. Varying degrees of mandibular overgrowth can be clinically detected in UCH patients. A classification in three categories has been described: hemimandibular elongation (HE), hemimandibular hyperplasia (HH) and a combination of these two (hybrid form) [2]. The asymmetrical development in UCH patients often results in functional and aesthetic problems [3]. No gold standard for diagnosis and treatment is available. (Hetero-) anamnesis in combination with clinical and radiological documentation and a positive SPECT-scan are currently being used to identify ongoing disease. Patients are considered to have hyperactivity of one condyle if the bone scintigram shows a >10% left to right difference [4], [5]. Treatment of these patients consists of removal of the growth center by a partial condylectomy. Secondly, correction of the facial asymmetry needs to be addressed, usually consisting of a combination of orthodontics and surgery [6]. Although the disease is self-limiting, asymmetry can become excessive. Especially in patients where the growth activity degree is hard to rate, for example when clinical evaluation indicates progression whereas the bonescintigraphy does not show a >10% right to left difference, accurate (imaging) documentation for monitoring is of utmost importance.

Facial asymmetry in patients with condylar hyperplasia has been subjectively described before, but an objective quantification is lacking [2], [6]. Objective quantification would offer possibilities to evaluate the development of the facial asymmetry in time. Secondly, it would offer a possibility to evaluate the effect and accuracy of treatment. With recent advances in 3D technology, objective quantification of facial asymmetries can be performed without the use of ionizing radiation or other invasive measures [7], [8].

The aim of this study was to objectively quantify facial and mandibular soft-tissue asymmetry in patients with unilateral condylar hyperplasia, and to evaluate whether this method is applicable for routine diagnostic and follow-up procedures. A new method based on 3D stereophotogrammetry to isolate the lower part of the face was validated and used to compare the patients to a control group.

## Materials and Methods

Thirty patients with proven unilateral condylar hyperplasia (UCH) and available 3D stereophotogrammetric images from September 2009 untill November 2011 were included in the study. UCH was defined using the following inclusion criteria: progressive mandibular asymmetry, supported with a positive bone-scan (difference in affected vs. non-affected region of interest >10%) and/or performed condylectomy. Exclusion criteria were proven mandibular fracture, previous mandibular surgery and facial asymmetry suspected to be based on a non UCH-cause. A control group of 30 age and gender matched healthy volunteers, without a prior history of facial surgery or existing facial deformities, was selected. This study was presented to the institutional review board of the VU University Medical Center, and it was decided that no ethical approval was needed, due to the retrospective and non-invasive nature. All patients were informed about the use of their photographs for research purposes besides the normal use for diagnosis and treatment. For all controls used in this study a written informed consent was obtained prior to photo acquisition and use. A consent protocol was developed and used. This procedure was discussed and approved by the ethics committee. The data were processed anonymously. The patient depicted in the article has given her written consent for publication.

For all patients and controls 3D photographs were acquired using a stereophoto-grammatrical camera set-up (3dMD face™ System, 3dMD, Atlanta, GA, USA). The 3D photographs were taken with the subject in a natural head position, eyes open and relaxed facial musculature [9]. All 3D photographs were taken by an experienced co-worker.

**Figure 1:**
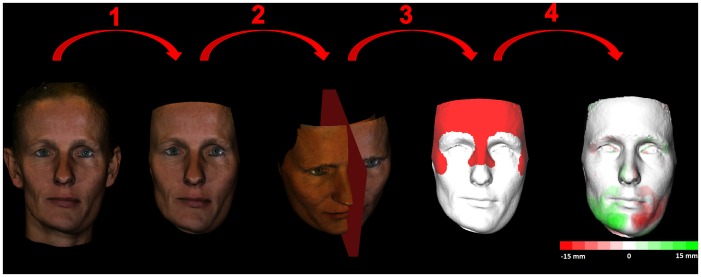
Illustrating step 1 removal of the confounding regions. Step 2 computing of a mirror image. Step 3 registration procedure using the selected areas. Step 4 creation of a distance map. (The individual in this photograph has given written informed consent (as outlined in PLOS consent form) to publish this picture).

**Figure 2:**
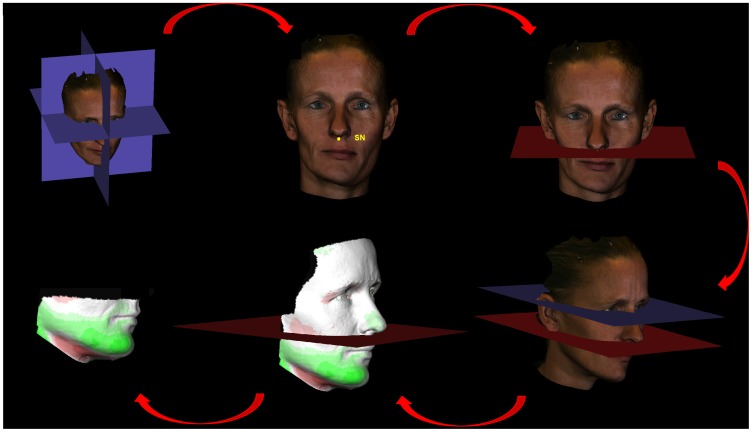
Illustrating step 5. A reference frame is set up. The subnasal landmark (Sn) is indicated through which a plane, perpendicular to the horizontal plane of the reference frame, is computed. The new plane is used to split the (in step 4 computed) distance map. (The individual in this photograph has given written informed consent (as outlined in PLOS consent form) to publish this picture).

Asymmetry quantification of the whole face was achieved using an existing method priorly published by Verhoeven et al. [10], which includes the following steps:

1. Using 3dMDpatient software the neck, ears and hair were removed to exclude confounding regions (3dMDpatient™ v3.1.0.3 Software Platform, 3dMD) (figure 1) [7].
2. In Maxilim® (Medicim NV, Mechelen, Belgium) a sagittal plane was constructed and used to create a mirrored 3D photograph (figure 1).
3. The original and the mirrored 3D photograph were matched using the Iterative Closest Point Algorithm [11]. This registration procedure was performed in Maxilim® using selected areas (forehead, upper nasal dorsum and zygoma [12]) (figure 1).
4. The registration procedure resulted in a color map which illustrates the distances between two corresponding points on both (original and mirrored) 3D photographs [13]. These distances were used as a direct measurement of the facial asymmetry. The absolute mean and the 95^th^ percentiles of the distances were calculated in millimeters using Matlab® (7.4.0 (R2007a) Mathworks, Natick, MA, USA) (figure 1).
5. As UCH is a mandibular disorder, most of the asymmetry is expected in this region. Separate evaluation of asymmetry in this particular area is desirable. Thus, a fifth step was added to isolate the lower part of the face.
6. The original photograph was imported into Maxilim® and a reference frame was set up [14]. The subnasal landmark was identified and a plane, parallel to the horizontal plane of the reference frame, was constructed through this landmark [15]. The new plane was used to remove the upper part of the distance map. Now the asymmetry of the lower face could be calculated (figure 2).

### Statistical Analysis and Validation

The described methods, for the complete and lower face, were applied to the patient and control group. The patients and controls were compared for the absolute mean and the 95^th^ percentile of the asymmetry. Significant differences (P<0.05) were tested using an unpaired Student’s T-test.

To investigate the inter-observer reproducibility of the lower face method, it was applied to the 3D photographs of five patients and five controls by two observers. To investigate the intra-observer error one of the observers repeated the measurements one week later. The absolute mean asymmetry and the 95^th^ percentile of the measurements were compared. A difference of less than 0.5 mm was considered clinically acceptable [14], [16]. The difference in means (95% confidence interval [CI]) and the standard error of the mean (SEM = SD/√N) were calculated to represent the systematic error. The measurement error (ME = SD/√2) was calculated to represent the random error.

### Categories

**Figure 3:**
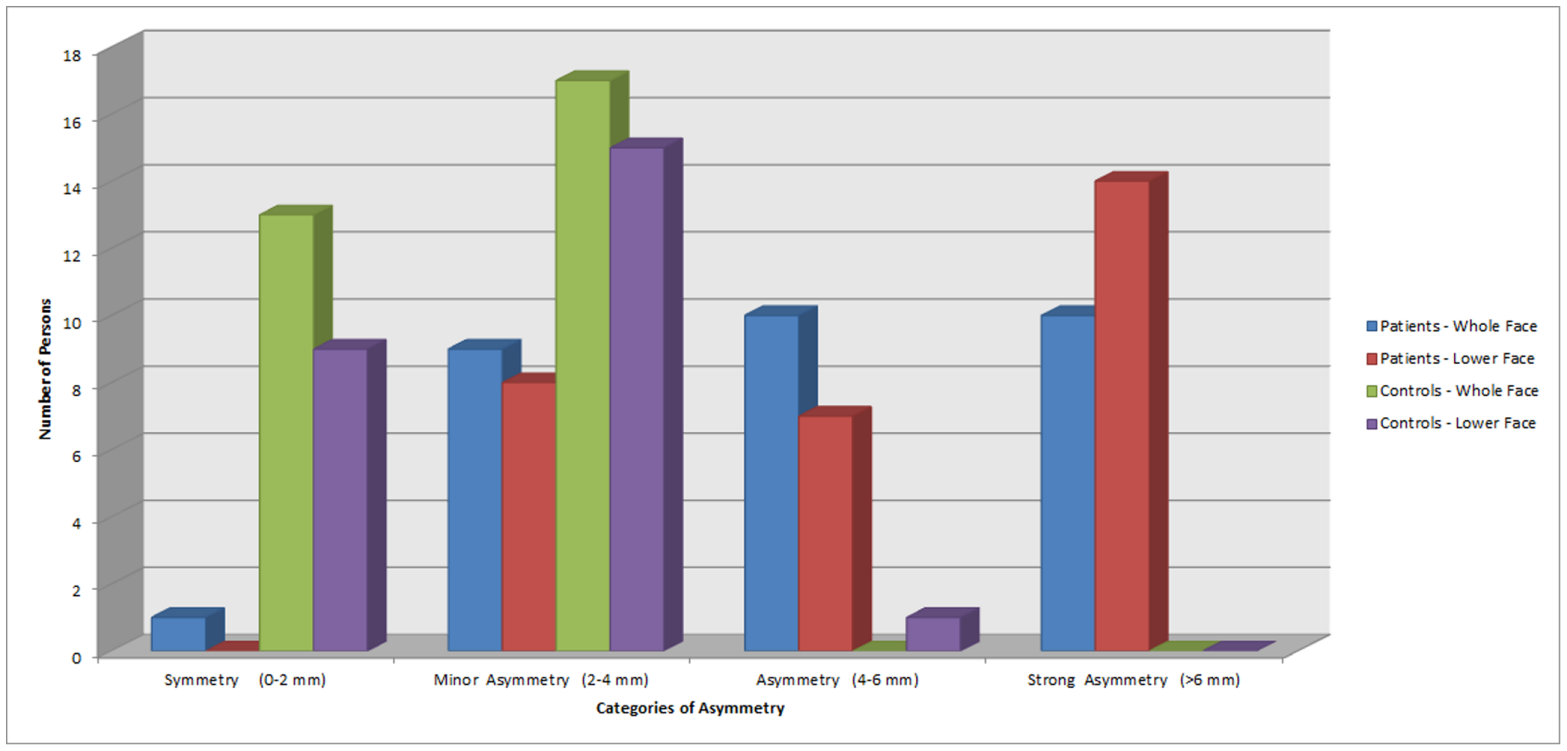
Histogram of the number of persons per category based on the 95^th^ percentile.

Apart from calculating the absolute mean and 95^th^ percentiles, the latter was used to divide all patients and controls into four categories (figure 3):

- symmetry (0–2 mm)
- minor asymmetry (2–4 mm)
- asymmetry (4–6 mm)
- strong asymmetry (>6 mm)

## Results

### Validation of the Lower Face Method

Table 1 shows the intra- and inter-observer performances of the lower face method. The intra-observer difference of the absolute mean asymmetry is 0.011 mm (−0.034–0.011) with a measurement error of 0.022 mm. The inter-observer difference is 0.017 mm (−0.007–0.042) with a measurement error of 0.024 mm.

**Table 1:** Intra- and inter-observer performances of the lower face method.

	Absolute mean			95^th^ percentile		
	Mean	SE	ME	Mean	SE	ME
Intra-observer	0.011	0.010	0.022	0.0054	0.007	0.015
(95%-CI)	(−0.034–0.011)			(−0.021–0.010)		
Inter-observer	0.017	0.011	0.024	0.010	0.008	0.017
(95%-CI)	(−0.007–0.042)			(−0.007–0.028)		

The results for the absolute mean and the 95^th^ percentile are shown. The difference in means (Mean) (95% CI) (mm), standard error (SE) (mm) and the measurement error (ME) (mm).

### Study

The study method was applied to 30 patients and 30 controls. The average age of the patient group was 22 years (±9, range 11–41 years) and included sixteen women and fourteen men.

The asymmetry for the complete face of both the patient and the control group is demonstrated in table 2. The absolute mean asymmetry in patients (1.57 mm) and controls (0.78 mm) showed a significant difference of 0.79 mm. In the 95th percentile of the asymmetry a significant difference (3.32 mm) between controls (2.12 mm) and patients (5.44 mm) was also found.

**Table 2:** The asymmetry of the whole face in patients and controls.

		Absolute mean	95^th^ percentile
Patients	Mean (mm)	1.57	5.44
	SD (mm)	0.62	2.59
Controls	Mean (mm)	0.78	2.12
	SD (mm)	0.20	0.57
Difference	Mean (mm)	0.79	3.32
	(95% CI)	(0.55–1.02)	(2.35–4.29)

For assessment of the lower face asymmetry two individuals in the patient group and five individuals in the control group had to be excluded because of overlying hair in the ear region, making it impossible to set up a reference frame. Therefore, the lower face asymmetry was measured for 28 patients and 25 controls. The results of the included subjects are presented in table 3. The absolute mean (2.64 mm vs. 1.01 mm) and 95^th^ percentile (6.47 mm vs. 2.29 mm) of the asymmetry both showed a significant difference between patients and controls of 1.63 mm and 4.18 mm respectively.

**Table 3:** The asymmetry of the lower face in patients and controls.

		Absolute mean	95^th^ percentile
Patients	Mean (mm)	2.64	6.47
	SD (mm)	1.35	2.97
Controls	Mean (mm)	1.01	2.29
	SD (mm)	0.40	0.74
Difference	Mean (mm)	1.63	4.18
	(95% CI)	(1.04–2.12)	(2.89–5.23)

## Discussion

Unilateral condylar hyperplasia is inextricably linked to facial asymmetry, most visible in the lower third of the face. This has been published on extensively, classifying two characteristical patterns: hemimandibular elongation and hemimandibular hyperplasia, and a third hybrid or mixed form of these two (HH/HE). HE exerts a horizontal asymmetry with a clear horizontal deviation of the chin. HH demonstrates a more vertical asymmetry with minor horizontal chinpoint deviation and/or cant. Usually regular photographs are taken to subjectively evaluate the asymmetry [2], [3], [17], [18], [19], [20]. To our knowledge objective 3D-quantification of the asymmetry has not been performed before. The aim of this study was to objectively quantify facial and mandibular soft-tissue asymmetry in patients with unilateral condylar hyperplasia, and to evaluate whether this method is applicable for routine diagnostic and follow-up procedures. A new method based on 3D stereophotogrammetry to isolate the lower part of the face was validated and used to compare the patients to a control group.

The used method is based on a 3D stereophotogrammetry system with a well-researched error of 0.1 mm and an acquisition time of 2 ms [21]. The small system error and fast acquisition time makes the system very suitable for quantifying soft-tissue facial asymmetry. Because the system is based on digital photography it is only a small burden and not invasive for the subject compared to radiographs including cone beam computed tomography. Digital photography is not able to image underlying bony structures and therefore is unable to assess the tissue-origin of asymmetry. Another limitation is the inability to capture the fine structures of the hair.

The study method to isolate and quantify mandibular asymmetry specifically, showed clinically acceptable intra- and inter-observer performance scores. The method is a modified version of the method described by Verhoeven et al. in 2012 [10]. The intra- and inter-observer performance scores (0.02 mm and 0.04 mm respectively) of the method described by Verhoeven et al. were mainly influenced by two manually performed steps in the procedure. The first is the removal of the confounding regions and secondly, the selection of the regions of interest for surface based registration. In this study the only variable was the indication of the subnasal landmark, as the other steps were already validated. This could explain the low intra- and inter-observer differences. An advantage for both the previously described method and the newly modified method is that it is not based on a facial midline but on surface based registration. The midline, especially in this patient group, does not naturally coincide with the facial symmetry axis [22]. Another advantage of the method is the possibility to measure asymmetry in the whole face independent of the direction of the asymmetry. This makes it applicable to various pathologies. In addition the analysis is easy and quick to use which makes it applicable to quickly measure asymmetry in a clinical setting.

Differences in the amount of asymmetry within one group and between the patient and control groups are illustrated using the 95^th^ percentile. Categories were made with a 2 mm difference, clearly visualizing the differences between the patient group and control group. All but one control are within the categories symmetry (0–2 mm) or minor asymmetry (2–4 mm). On the contrary, all but one of the patients are within the three asymmetric categories. The patients whole face asymmetry is equally distributed over the three categories. While the patients lower face asymmetry has a striking peek in the strong asymmetry (>6 mm) category (figure 3). By categorizing patients in different asymmetry groups, a systematic approach to diagnosis, treatment and follow-up would become possible. Hwang et al. performed a classification of facial asymmetry by cluster analysis, using measurements on frontal cephalograms and photographs [23]. According to the results 100 patients were divided in five asymmetry subgroups. Each group appeared to have a specific etiology and different treatment modality. The classification system proved to be of great help in accurate diagnosis and treatment planning of facial asymmetries. The four categories in this study show a severity of asymmetry and do not differentiate in location of asymmetry (such as elongation or hyperplasia). Secondly, there is no discrimination in origin (such as mandibular asymmetry or muscular/soft tissue hyperplasia). However, these categories in severity could be a prognostic factor for treatment, and could lead to earlier intervention in patients that at first presentation are already scheduled in category four.

In 2010, Meyer-Marcotty et al. compared subjective ratings of pictures with objective measurements of asymmetry [24]. A 3D optical sensor was used and asymmetry was calculated by mirroring, surface based registering and calculating the average absolute mean distance between the original and the mirrored 3D surfaces. Eighteen unilateral cleft lip and palate patients were compared with eighteen random control persons. A significant difference in whole face asymmetry (patients 0.87 mm - controls 0.59 mm) as well as a positive correlation between objective asymmetry and appearance rating were found. Apart from the whole face asymmetry they isolated the lower face (subnasale to gnathion). The lower faces had a mean asymmetry of 0.79 mm in patients and 0.59 mm in controls. These asymmetries are rather small compared to this study. Part of this difference can be explained by the difference in pathology. Condylar hyperplasia is expected to result in more overall facial asymmetry than cleft lip and palates. This is especially clear in the patients’ lower face regions 2.59 mm (this study) vs. 0.79 mm (Meyer-Marcotty) of asymmetry. Part of the difference might also be explained by the exclusion of confounding regions. This was not described by Meyer-Marcotty et al. If the excluded regions contain more of the facial asymmetry it will not be taken into account in the mean facial asymmetry measurement and therefore result in a lower mean for both controls and patients. Furthermore, the difference in isolating the lower face might also influence the outcome. Meyer-Marcotty et al. described the method of isolation only in 2D and no validation study was reported.

In 2009 and 2011, Primozic et al. studied the correction of unilateral posterior crossbite in the primary dentition using an acrylic plate expander [25], [26]. Two Konica/Minolta Vivid 910 laser scanners were used for image acquisition. The images were mirrored, surface based registered and the absolute mean distance between surfaces was calculated as a measure of asymmetry. Facial images of 30 children with a unilateral posterior crossbite (with at least 2 mm midline deviation) and 30 without malocclusion were compared. The whole face asymmetry was compared and no significant difference in asymmetry was found between patients and controls. The whole face asymmetry was found to be 0.50 mm for patients and 0.44 mm for controls. These are small asymmetries compared to the results in this study on condylar hyperplasia. The difference can again be explained by the different pathologies and the possible difference in exclusion of confounding regions. The authors mentioned the removal of unwanted data, but they did not exactly describe which data.

In a previous study by Verhoeven et al. patients with mandibular reconstruction were compared with an age and gender matched control group [10]. For the whole face asymmetry measurement, the same method as in this study was used. For the lower face asymmetry measurement a non-validated plane through three landmarks (subnasal, left and right alar curvature) was used. Significant differences were found between patients and controls for both whole (2.21 mm vs 1.02 mm) and lower face asymmetry (3.37 mm vs 1.25 mm). The larger results compared to this study can be partially explained by the different pathologies. But these differences do not explain the difference in the whole face control group of both studies (0.78 mm (this study) vs. 1.02 mm (Verhoeven [10]) which was done with the exact same measurement method. A possible explanation is the difference in age between the control groups: mean age of 22 years (range 11–41) vs. 54 years (range 15–74). This leads to the presumption that with ageing facial asymmetry increases. This is an interesting hypothesis for further studies.

Time is an important factor in progressive disorders such as UCH, and is being referred to as “the fourth dimension”. Kaban describes progression of deformity with time in mandibular asymmetry as a result of undergrowth and overgrowth, and states that understanding this process is the basis for diagnosis and treatment [27]. Although UCH is a self-limiting disease, prevention of end-stage gross deformities is crucial, and development of secondary midfacial deformities should be avoided. With increasing severity of asymmetry, surgical correction becomes more extended and usually has to be performed bilateral and bimaxillary. Prevention of this could be performed by in time removal of the abnormal growth center with a partial condylectomy. Thus, identifying progression of the disease at time of diagnosis is of utmost importance for further treatment planning. Establishing the presence of progression is tenuous, history-taking, earlier documentation of photographs, radiographs and even bone-scans are of relative importance since no gold standard is available. This study demonstrates that 3D sterophotogrammetry is a useful tool for quantification of overall facial and lower facial asymmetry.

With a substantial database of patients with unilateral condylar hyperplasia, it might be interesting to perform a more extensive study on follow-up from the moment of established diagnosis until the moment of finalizing treatment.

### Conclusion

There is a significant difference in facial and mandibular soft-tissue asymmetry between patients with unilateral condylar hyperplasia and controls. The new method used to isolate mandibular asymmetry proved to be valid and is a suitable tool to produce a more in-depth evaluation of asymmetry of the lower face. 3D stereophotogrammetry is easily applicable for routine diagnostic procedures and seems useful for follow-up of UCH patients.
